# Retina image segmentation using the three-path Unet model

**DOI:** 10.1038/s41598-023-50141-0

**Published:** 2023-12-19

**Authors:** Ruihua Liu, Wei Pu, Haoyu Nan, Yangyang Zou

**Affiliations:** 1 School of Artificial Intelligence, Chongqing University of Technology, Chongqing, China; 2 Chongqing Vocational College of Transportation, Chongqing, China; 3 OPT Machine Vision Tech Co., Ltd., Guangdong, China

**Keywords:** Oncology, Biomedical engineering

## Abstract

Unsupervised image segmentation is a technique that divides an image into distinct regions or objects without prior labeling. This approach offers flexibility and adaptability to various types of image data. Particularly for large datasets, it eliminates the need for manual labeling, thereby it presents advantages in terms of time and labor costs. However, when applied to retinal image segmentation, challenges arise due to variations in data, presence of noise, and manual threshold adjustments, which can lead to over-segmentation or under-segmentation of small blood vessel boundaries and endpoints. In order to enhance the precision and accuracy of retinal image segmentation, we propose a novel image supervised segmentation network based on three-path Unet model.Firstly, the Haar wavelet transform is employed to extract high-frequency image information, which forms the foundation for the proposed HaarNet, a Unet-inspired architecture. Next, the HaarNet is integrated with the Unet and SegNet frameworks to develop a three-path Unet model, referred to as TP-Unet. Finally, the model is further refined into TP-Unet+AE+DSL by incorporating the advantages of auto-encoding (AE) and deep supervised learning (DSL) techniques, thereby enhancing the overall performance of the system. To evaluate the effectiveness of our proposed model, we conduct experiments using the DRIVE and CHASE public datasets. On the DRIVE dataset, our recommended model achieves a Dice coefficient of 0.8291 and a sensitivity index of 0.8184. These results significantly outperform the Unet model by $1.34\%$ and $2.60\%$, respectively. Furthermore, our model demonstrates excellent performance on the CHASE dataset, with a Dice coefficient of 0.8162, a sensitivity of 0.8242, and an accuracy of 0.9664. These metrics surpass the Unet model by $3.20\%$, $6.66\%$, and $0.42\%$, respectively. Our proposed model provides more accurate and reliable results for retinal vessel segmentation, which holds significant potential for assisting doctors in their diagnosis.

## Introduction

Fundus vascular image segmentation technology plays a crucial role in the diagnosis of various eye diseases, such as macular degeneration, atherosclerosis, diabetic retinopathy, glaucoma, and stroke^1^. Through accurate segmentation and analysis of blood vessels, medical professionals can easily identify abnormal vascular morphology and other lesions, thereby facilitating early diagnosis and treatment. The precise segmentation of fundus vascular images is essential for analyzing morphological changes in blood vessels, including alterations in vessel width, vessel density, and vessel branching patterns. The manual labeling of diseased vessels by experienced experts is a laborious and time-consuming task, highlighting the significance of automatic segmentation methods for ocular vascular images.

**Figure 1:**
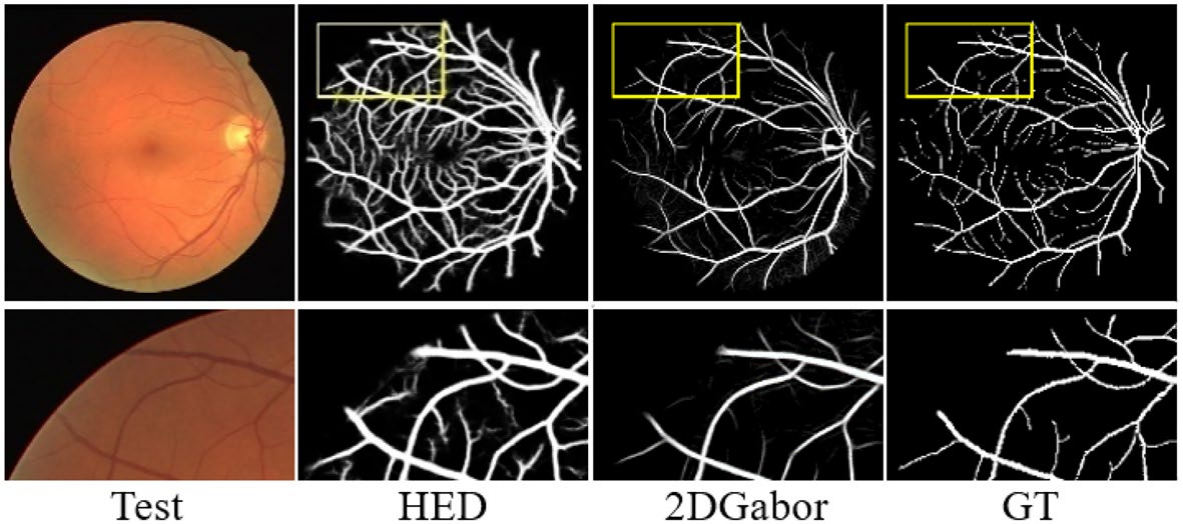
Comparative evaluation of unsupervised learning segmentation algorithms.

Currently, image segmentation algorithms can be broadly classified into two categories. One category comprises unsupervised segmentation algorithms, such as the matched filter method^2^, multi-threshold-based vessel detection^3^, boundary detection-based segmentation method^4^, 2D Gabor wavelet segmentation method^5^, morphology-based extraction method^6^, fuzzy clustering-based segmentation method^7^, and wavelet K-means clustering and Fuzzy method^8^. Unsupervised segmentation algorithms offer the advantage of achieving retinal vessel segmentation without the need for manual labeling by experts. In fundus or retinal images, small blood vessels are often difficult to identify with the naked eye. By utilizing computer-aided techniques to enhance and segment these small blood vessels, doctors can obtain clearer visibility. This is particularly important for the early detection and monitoring of disease progression, as initial symptoms of certain eye diseases may be concealed within these tiny blood vessels. Unfortunately, these methods still possess certain limitations. For instance, the HED method often produces fuzzy noise blocks during vessel segmentation, leading to more noticeable over-segmentation. On the other hand, the Gabor wavelet-based feature extraction method encounters challenges in under-segmentation, resulting in the omission of some tiny vessels, as depicted in Fig. 1.

Another category of segmentation algorithms is supervised segmentation algorithms. These algorithms primarily rely on data-driven approaches to learn and enhance their ability to segment images^9–13^. For instance, Long et al.^9^ proposed an end-to-end fully convolutional network (FCN) model for semantic segmentation. Ronneberger et al.^10^ recommended the use of the Unet model, which employs an encoder-decoder architecture, for medical image segmentation. Alom et al.^11^ introduced a recursive residual network based on Unet to improve the feature representation of retinal vascular images and enhance segmentation accuracy. Guo et al.^12^ demonstrated the effectiveness of a GAN-DenseNet model, which combines adversarial network modeling and a densely connected structure, in optimizing the segmentation network. Additionally, Zou et al.^13^ presented a multi-label local regression method to guide the CNN model in generating complete retinal vascular images.

To enhance image quality and contrast, Schmidt et al.^14^ utilized the contrast limited adaptive histogram equalization (CLAHE) method and a median filter. Bukenya et al.^15^ developed a 2D hybrid multi-scale method for blood vessel segmentation, incorporating the White Top-Hat scale-space Bilateral Hessian Vessel Enhancement Filter, the Hysteresis threshold method, and the MATLAB bwareaopen operation, resulting in improved segmentation outcomes.

While these deep learning-based segmentation algorithms have improved the accuracy of retinal vessel segmentation to some extent, they face challenges when dealing with fine vessels and subtle differences between vessel boundaries and background pixels. This can lead to feature information loss during the extraction process and limit the performance of the segmentation models.

To address these limitations, we propose a three-path Unet segmentation model, called TP-Unet, that incorporates the advantages of Haar wavelet transform for extracting richer feature information. Haar wavelet transform is a widely used feature extraction method that captures low-frequency and high-frequency information in different directions.

The concept of auto-encoder (AE) was introduced by Hinton et al. in 2006^16^. In 2021, Baur et al.^17^ incorporated the auto-encoder branch into the Unet model, thereby enhancing the ability of the low-resolution coding layer to express feature information. Furthermore, in 2020, Liu et al.^18^ introduced the concept of deep supervision learning (DSL) in the decoding stage, improving both the convergence speed of the model and its ability to capture fine-grained features.

The TP-Unet+AE+DSL model, which incorporates the TP-Unet model, the AE block, and the DSL block, offers a viable solution to address the issue of under-segmentation. Herein, we present a concise overview of the key contributions of our work.

- To enhance the extraction of spatial semantic features, preserve greater boundary detail, and obtain more comprehensive frequency domain features, a novel hybrid training framework, denoted as TP-Unet, has been designed. This framework synergistically integrates the capabilities of Unet, SegNet, and HaarNet architectures. The main purpose of TP-Unet is to strengthen the boundaries of tiny vessel segmentation, thereby improving the accuracy of retinal image segmentation.
- Building upon the TP-Unet model, we have developed an advanced fusion model, the TP-Unet+AE+DSL model. This model leverages the AE module’s ability to characterize low-resolution features and the DSL module’s capacity to gather intricate information. Through this combination, our model achieves superior performance.
- We conduct rigorous testing on two publicly available datasets, namely the DRIVE^19^ and CHASE^20^ datasets. The results demonstrate that our model exhibits higher accuracy and greater comprehensiveness compared to alternative approaches.

**Figure 2:**
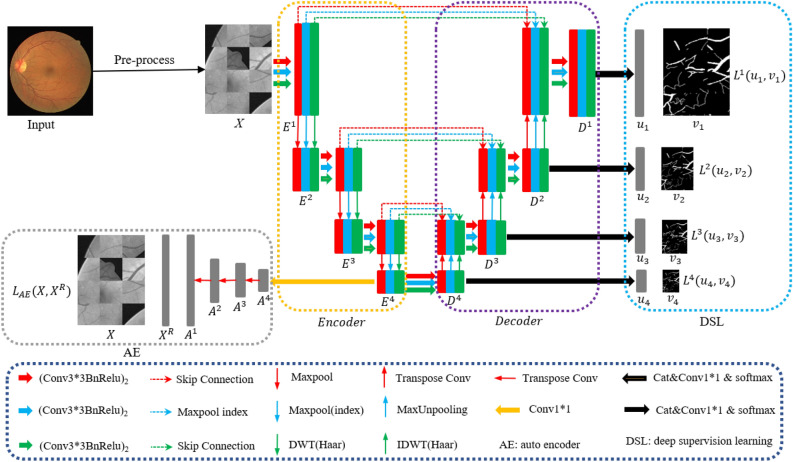
TP-Unet+AE+DSL framework.

## Method

The model architecture comprises three components: the TP-Unet model, the AE block, and the DSL block, as depicted in Fig. 2. The TP-Unet model employs a three-path fusion structure, integrating the Unet^10^ model, SegNet^21^ model, and Haar wavelet decomposition model, which enables the extraction of frequency domain features from the image. The purpose of the AE block is to enhance the representation of the auto-encoder layer features, while the DSL block aims to minimize the loss of fine-grained features. Consequently, the network model can be represented as follows,

1 $$\begin{aligned} f(X)=[u_1, u_2, u_3, u_4, X^R], \end{aligned}$$

where $X \in {\mathbb {R}}^{1 \times W \times H }$ is the input data, $u_1, u_2, u_3$ and $u_4$ are the output of each decoder layer, $u_i \in {\mathbb {R}}^{ 2 \times W/2^{i-1} \times H/{2^{i-1}} }, i = 1,2,3$. $X^R \in {\mathbb {R}}^{1 \times W \times H }$ is the output of the auto-encoder reconstruction, and $[u_1, u_2, u_3, u_4, X^R]$ is the output of the training set. The model parameters are updated by the loss function. The output $u_1 \in {\mathbb {R}}^{2 \times W \times H }$ is the prediction probability graph in the model prediction, where *W*, *H* is the width and height of the image, respectively.

### TP-Unet

#### Haar wavelet

Haar function $\varphi (x)$ is defined as,

2 $$\begin{aligned} \varphi (x)=\left\{ \begin{array}{ll} 1, &{}\quad 0 \le x \le 1, \\ 0,&{}\quad \text{ else } . \end{array}\right. \end{aligned}$$

The Haar wavelet formula is expressed as $\psi (x)=\varphi (2 x)-\varphi (2 x-1)$. We define the space $V_j$ as the sum of all $a_k$ multiplied by $\varphi (2^j x-k)$, where $a_k$ belongs to the set of real numbers. Additionally, we have the inclusion relationship $V_0 \subset V_1 \subset \cdots \subset V_j \subset \cdots$, where *j* takes on values in the set of non-negative integers. Moreover, we define the space $W_j$ as the sum of all $d_k$ multiplied by $\psi (2^j x-k)$, where $d_k$ is a real number. It is worth mentioning that $V_{j+1}$ is obtained by taking the direct sum of $W_j$ and $V_j$.

In summary, the image space can be described as follows:

3 $$\begin{aligned} V_j=V_0+W_0+W_1+\mathrm { \cdots } W_{j-1}. \end{aligned}$$

**Figure 3:**
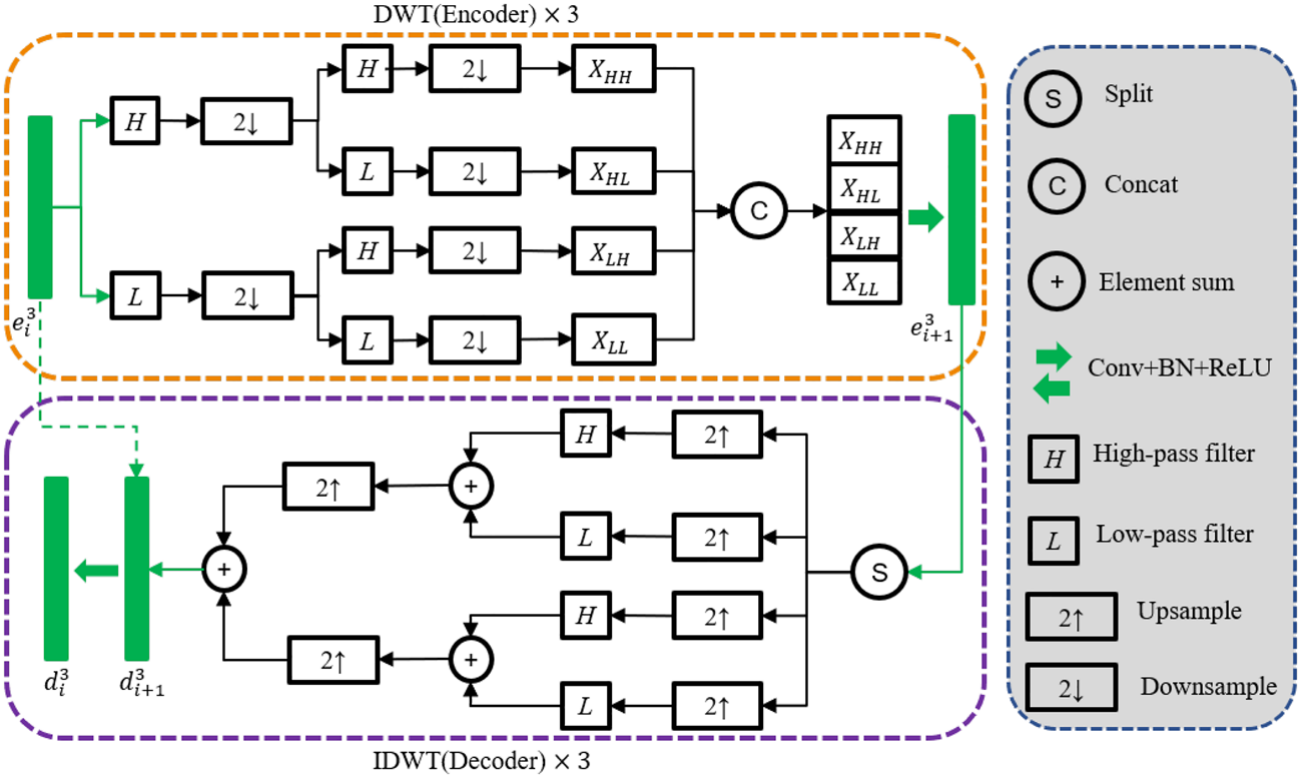
HaarNet model.

As we known, the larger *j* is, the more complete $V_j$ is, which equals to the more detailed information contained in. In order to address the limitations of the existing Unet network, which exhibits inadequate capability in extracting features from retinal blood vessel images and low accuracy in segmenting fine blood vessels, we propose the integration of Haar wavelet decomposition into the encoder-decoder architecture of the ’U’ shaped network, referred to as the ’HaarNet’ model. This novel model will be combined with the Unet model to enhance the feature extraction capability for fine vessels (see Fig. 3).

The HaarNet model initially decomposes the feature map using Haar wavelets, resulting in the acquisition of low-frequency features $X_{LL}$, horizontal high-frequency features $X_{HL}$, vertical high-frequency features $X_{LH}$, and diagonal high-frequency features $X_{HH}$. Subsequently, a $3 \times 3$ convolution kernel is employed to capture both the low-frequency subject information and high-frequency detail information of the image. Finally, the image is reconstructed using the wavelet reconstruction algorithm to restore the initial resolution (see Fig. 3).

The HaarNet model process can be expressed as follows,

4 $$\begin{aligned} g_i=\text {CBR}_2(e_i^3), \end{aligned}$$

5 $$\begin{aligned} e_{i+1}^3=\text {Concat}[\text {DWT}\left( g_i\right) ], \end{aligned}$$

6 $$\begin{aligned} g_i^{\prime }=\text {IDWT}[\text {Split}(d_{i+1}^3)], \end{aligned}$$

7 $$\begin{aligned} d_i^3=\text {CBR}_2[\text {Concat}(g_i^{\prime }, g_i)], \end{aligned}$$

where $e_1^3=X, d_4^3=\text {CBR}_2(e_4^3), i=1,2,3$, and $\text {CBR}_2(\cdot )$ is the operation of performing convolution, batch normalization and ReLU (CovBNReLU) twice.

**Figure 4:**
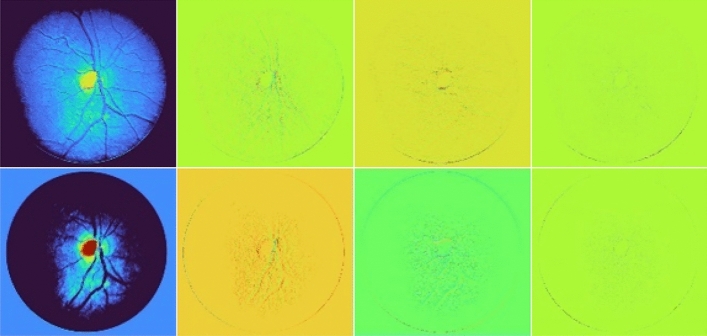
Visualization of the feature map in the HaarNet model. Columns 2–4 depict the high-frequency information.

The visualization of the feature map of the HaarNet model is presented in Fig. 4. It is notable that the high-frequency features exhibit a greater level of detailed information in the blood vessels, thereby enhancing the accuracy of segmenting the smaller vessels.

#### SegNet module

The SegNet semantic segmentation model, which was demonstrated by Badrinarayanan et al. in 2017^21^, utilizes the pooling index encoder-decoder strategy. This approach involves recording the maximum pooled pixel position index during the encoder stage and passing this index value to the decoder stage to aid in upsampling. By doing so, the model not only reduces computation and significantly improves network operation speed, but also better preserves the edge information of the image. In order to optimize the segmentation effect on vessel boundaries, we propose the construction of a pooled index encoder-decoder structure to enhance the segmentation performance.

**Figure 5:**
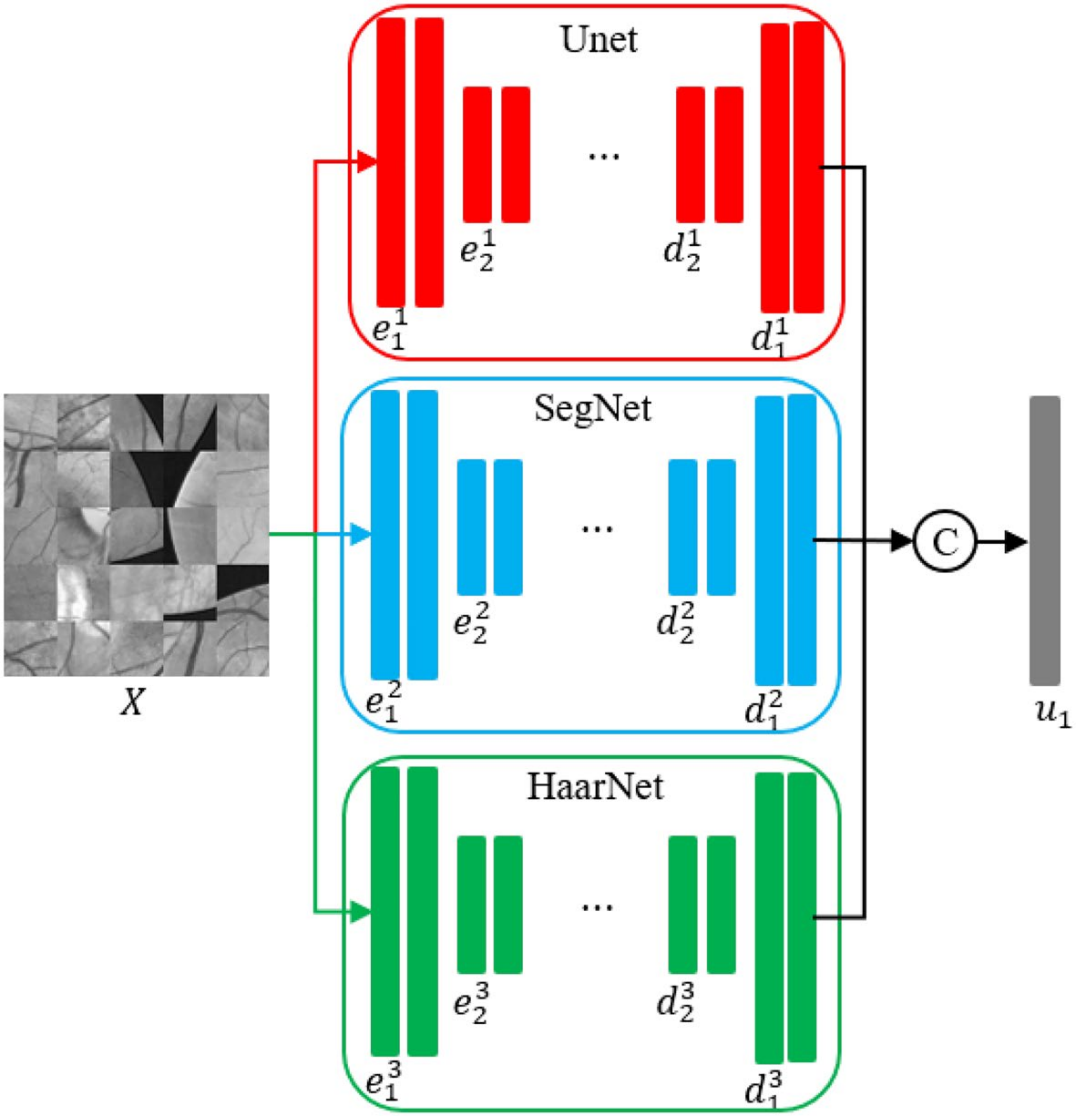
TP-Unet model.

#### Three-path fusion model

In order to explore the advantages of the Unet model, the SegNet model, and the HaarNet model, we propose a novel fusion model called the TP-Unet model. This model aims to improve the segmentation capability and accuracy of retinal vascular images, as depicted in Fig. 5. The TP-Unet model incorporates three distinct branches: the Unet branch, responsible for extracting spatial features along the red path; the SegNet branch, which enhances boundaries and endpoints along the blue path; and the Haar wavelet branch, which captures frequency domain features along the green path. By combining these three feature extraction methods, the TP-Unet model demonstrates enhanced performance. Please refer to Algorithm 1 for further details.

**Algorithm 1:**
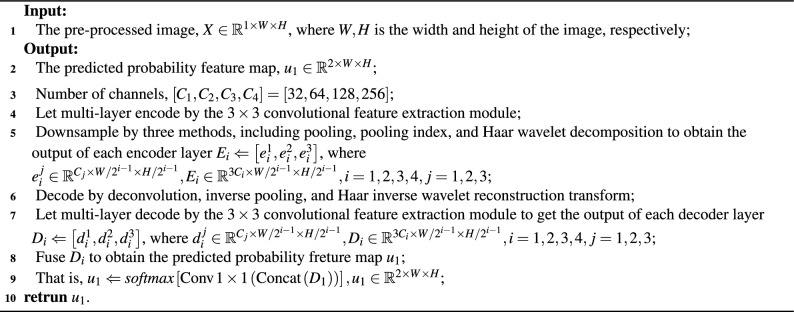
TP-Unet fusion architecture.

### AE module

The TP-Unet model demonstrates the capability to capture various features, including spatial features, boundary details, and frequency domain information. This ability proves to be highly advantageous in enhancing the accuracy of retinal vessel segmentation. Additionally, the AE block, functioning as an unsupervised learning structure, is proposed to be integrated into the TP-Unet model. This integration aims to optimize the feature representation capability of the encoding process.

The mapping function $f_E: X \rightarrow E_4$ is utilized to represent the transformation from the input image *X* to the final encoder layer $E_4$, denoted as $E_4 = [e_4^1, e_4^2, e_4^3 ]$. Subsequently, the $E_4$ features are combined and the number of channels is adjusted through a $1 \times 1$ convolution operation, resulting in the reconstructed output $A_4 = \text {Conv} 1 \times 1[\text {Concat}(E_4)]$. Two approaches are employed in the reconstruction process. Firstly, the initial resolution is restored iteratively through multiple deconvolution operations, yielding $A_1$. Secondly, $X^R=\text {Conv} 1 \times 1(A_1)$ is reconstructed by adjusting the channels and implementing the AE block. Thus, the mapping function from $E_4$ to $X^R$ is represented by $f_A: E_4 \rightarrow X^R$.

To minimize the discrepancy between the input image *X* and the reconstructed output features $X^R$, and to enhance the feature representation in the TP-Unet model encoder, the mean squared error (MSE) loss function is employed in the self-encoder.

8 $$\begin{aligned} \begin{aligned} {\text {Loss}}_{A E}&=\Vert X-f_A[f_E(X)]\Vert ^2 \\&=\frac{1}{n} \sum _{j=1}^n|X(j)-X^R(j)|^2. \end{aligned} \end{aligned}$$

### DSL module

The GoogLeNet model, as recommended by Szegedy et al.^22^, has been widely recognized for its effectiveness in deep supervised learning. This model successfully addresses the issues of gradient disappearance and slow convergence that are commonly encountered in traditional deep learning mechanisms. In comparison to these traditional approaches, deep supervised learning proves to be more efficient in enhancing the feature extraction capability by incorporating auxiliary branching loss functions at various training stages. Furthermore, to mitigate the potential loss of fine-grained features caused by cascading convolution and nonlinear transformations, a DSL module is implemented in each decoder layer of the TP-Unet model, as depicted in Fig. 2.

Let $D_i=[d_i^1, d_i^2, d_i^3 ]$ denote the decoder for each layer. These three-path decoders are combined and the number of channels is adjusted using a $1 \times 1$ convolution kernel. The predicted probability map for each layer decoder output, denoted as $u_i$, is obtained using the softmax activation function applied to the result of concatenating $D_i$ and passing it through a $1 \times 1$ convolution layer, i.e., $u_i=softmax [\text {Conv1} \times 1(\text {Concat}(D_i))]$. Here, $u_i$ is in the range of [0, 1] and *i* ranges from 1 to 4. Finally, the model parameters are optimized by calculating the error between the predicted probability maps and the vessel label samples using a multi-path branching loss function.

9 $$\begin{aligned} \text {Loss}_{DSL}=\sum _{i=1}^4 \alpha _i L^i(u_i, v_i). \end{aligned}$$

The symbols $\alpha _i$, $L^i(u_i, v_i)$ represent the equilibrium coefficient of the loss function and the cross-entropy loss function for each branch, where *i* takes values from 1 to 4.

## Experiments

### Datasets and evaluation metrics

In this paper, we conduct experiments on two public datasets, namely DRIVE^19^ and CHASE^20^. Each dataset comprises two sets of labels obtained from two different observers, with the first observer considered as the ground truth (GT). The DRIVE dataset is a commonly used dataset for retinal vessel segmentation, consisting of a total of 40 labeled retinal vessel images, each with a resolution of $565 \times 584$. The first 20 images are utilized as the training set, while the remaining 20 images are designated as the test set. On the other hand, the CHASE dataset consists of 28 retinal images captured from the eyes of 14 school children, with each image having a resolution of $999 \times 960$. For the CHASE dataset, the first 20 images are selected as the training set, while the last 8 images are assigned to the test set.

During the experiment, we employ TP (true positive), FP (false positive), FN (false negative), and TN (true negative) as the indicators for evaluation. Specifically, we calculate the Dice coefficient, sensitivity, specificity, and accuracy, which are presented in Table 1.

**Table 1:** Evaluation metrics.

Evaluation metrics	Abbr.	Calculation formula
Dice coefficient	Dice	2TP/(2TP+FP+FN)
Sensitivity	SE	TP/(TP+FN)
Specificity	SP	TN/(FP+TN)
Accuracy	Acc	(TP+TN)/(FP+FN+TP+TN)

### Preprocessing and implementation

Due to the influence of uneven illumination, fundus photos require preprocessing. The first step involves converting the color fundus photos into grayscale.

10 $$\begin{aligned} X=0.299 R+0.578 G+0.114 B. \end{aligned}$$

The retinal dataset used for training purposes has a relatively small sample size. To address this limitation and enhance the diversity of the training set, various preprocessing techniques are applied to both the images and labels. These techniques include grayscale conversion, flipping, rotation, horizontal and vertical flipping, as well as translational transformations. Consequently, the training set is expanded by incorporating these techniques.

**Figure 6:**
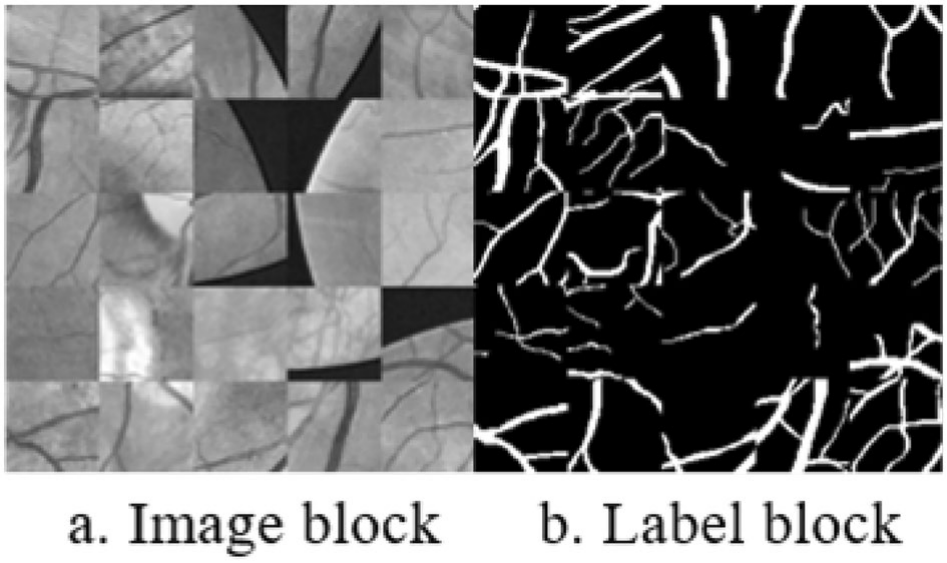
Preprocessed block.

Following the expansion of the training set, 4000 images of a specified size of $64 \times 64$ are randomly selected through a cropping process. This process is visually represented in Fig. 6.

The total loss function used for model training is as follows:

11 $$\begin{aligned} \begin{aligned} {\text {Loss}}&=\beta \cdot \text {Loss}_{AE}+\text {Loss}_{DSL} \\&=\beta \cdot L_{AE}(X, X^R)+\sum _{i=1}^4 \alpha _i \cdot L^i(u_i, v_i). \end{aligned} \end{aligned}$$

In this study, we consider the equilibrium coefficients of the AE module, denoted as $\beta$ and $L_{AE}(X, X^R)$, as well as the MSE loss function. Additionally, we examine the equilibrium coefficients of each branch loss function, represented by $\alpha _i$ and $L^i(u_i, v_i)$, where *i* ranges from 1 to 4. Moreover, we analyze the cross-entropy loss function. For the purpose of model training, we set the equilibrium coefficients as follows: $[\beta , \alpha _1, \alpha _2, \alpha _3, \alpha _4]=[0.1,1,0.2,0.2,0.2]$.

This paper presents the configuration of the experimental environment, which includes the utilization of an Intel(R) Core(TM) i7-10750H CPU, an NVIDIA RTX 2070 Super GPU, 8GB of video memory, and 16GB of system memory. The experiments are conducted using the PyTorch framework, employing a batch size of 64 and training for a total of 50 epochs. The optimization process is performed using the Adam algorithm, initialized with a learning rate of 0.0005.

### Evaluate metric comparisons

In order to assess the effectiveness of the proposed TP-Unet+AE+DSL model, a series of experiments are conducted, comparing its performance with other advanced methods using the DRIVE^19^ dataset and CHASE^20^ dataset.

**Table 2:** Comparison of evaluation metrics using advanced methods on the DRIVE dataset.

Model	Year	Dice	SE	SP	Acc
Unet^10^	2015	0.8157	0.7924	0.9781	0.9544
Bukenya et al.^15^	2018		0.7490	0.9740	0.9540
ReUnet^11^	2019	0.8155	0.7751	0.9816	0.9556
R2Unet^11^	2019	0.8171	0.7792	0.9813	0.9556
Zou et al.^13^	2021	0.8129	0.7761	0.9792	0.9519
Du et al.^23^	2021		0.7814	0.9810	0.9556
ContextUnet^24^	2021	0.8135	0.7961	0.9778	0.9547
CSUnet^24^	2021	0.8251	0.8071	0.9782	0.9565
Tariq et al.^25^	2022		0.8125	0.9763	0.9610
Li et al.^26^	2022	0.7994	0.7882	0.9815	**0.9657**
LSW-Net^27^	2022	0.8216	0.7876	**0.9837**	0.9565
ResDO-Unet^28^	2023	0.8229	0.7985	0.9791	0.9561
Wang et al.^29^	2023	**0.8687**	0.7380	0.9703	0.9403
**Ours**		0.8291	**0.8184**	0.9773	0.9571

Significant values are in bold.

The evaluation on the DRIVE dataset involves the comparison of our model against 13 existing methods, namely Unet^10^, Bukenya et al.^15^, ReUnet^11^, R2Unet^11^, Zou et al.^13^, Du et al.^23^, ContextUnet^24^, CSUnet^24^, Tariq et al.^25^, Li et al.^26^, LSW-Net^27^, ResDO-Unet^28^, and Wang et al.^29^. Several evaluation metrics, including sensitivity, dice coefficient, and accuracy, are utilized for the purpose of comparison. Table 2 presents that our model exhibits optimal sensitivity. Furthermore, our model’s Dice coefficient is surpassed only by that of Wang et al.^29^, indicating a high level of performance. However, in terms of accuracy, our model ranks slightly lower, with only the work of Li et al.^26^ achieving superior results.

Similarly, on the CHASE dataset, our model is compared against 12 advanced methods, namely Unet^10^, ReUnet^11^, R2Unet^11^, Jin et al.^30^, Li et al.^31^, Jiang et al.^32^, Samuel et al.^33^, Yang et al.^34^, Tariq et al.^25^, ResDO-Unet^28^, Wang et al.^29^, and Hu et al.^35^. The results, as depicted in Table 3, indicate that our model exhibits optimal performance in terms of the Dice coefficient, sensitivity, and accuracy.

**Table 3:** Comparison of evaluation metrics using advanced methods on the CHASE dataset.

Model	Year	Dice	SE	SP	Acc
Unet^10^	2015	0.7842	0.7576	0.9826	0.9622
ReUnet^11^	2019	0.7928	0.7756	0.9820	0.9634
R2Unet^11^	2019	0.7810	0.7459	0.9836	0.9556
Jin et al.^30^	2019	0.7883	0.8155	0.9752	0.9610
Li et al.^31^	2020	0.8073	0.8073	0.9823	0.9655
Jiang et al.^32^	2021		0.7818	0.9819	0.9638
Samuel et al.^33^	2021		0.7233	**0.9865**	0.9633
Yang et al.^34^	2021	0.7997	0.8176	0.9776	0.9632
Tariq et al.^25^	2022		0.8012	0.9730	0.9578
ResDO-Unet^28^	2023	0.8236	0.8020	0.9794	**0.9672**
Wang et al.^29^	2023	**0.8358**	0.7413	0.9720	0.9504
Hu et al.^35^	2023	0.7922	0.7822	0.9830	0.9652
**Ours**		0.8162	**0.8242**	0.9805	0.9664

Significant values are in bold.

Upon analyzing both Tables 2 and 3, it becomes evident that our model exhibits a lower specificity index in both the DRIVE^19^ dataset and CHASE^20^ datasets. This suggests that our model has a higher recognition rate for retinal vascular pixels, while simultaneously reducing the risk of misidentifying blood vessel pixels as background pixels.

Furthermore, it is important to note that our model achieves the highest Dice coefficient score, highlighting its overall effectiveness in both retinal blood vessel segmentation and background pixel identification. This further emphasizes the high accuracy of our model in the segmentation of retinal blood vessel images.

### Segmentation visualization

**Figure 7:**
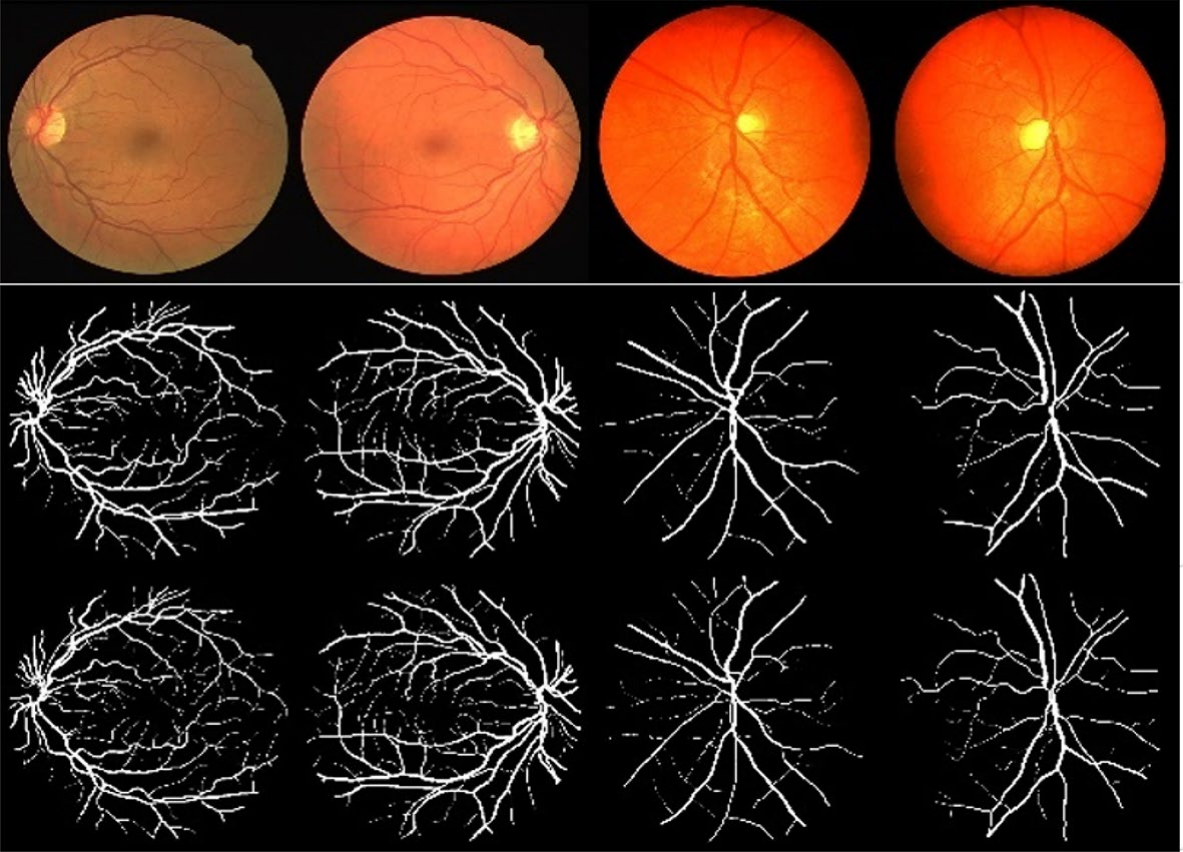
Visualization of segmentation using our model. The samples in columns 1–2 represent the DRIVE dataset, while the samples in columns 3–4 represent the CHASE dataset. The rows 1–3 correspond to the Test, Ours, and GT (Ground Truth) images, respectively.

Figure 7 illustrates the visualization results of our model’s segmentation on both the DRIVE^19^ dataset and the CHASE^20^ dataset. Comparing our model’s results with the reference standard, it is evident that our model successfully distinguishes the vascular region from the background region, displaying more complete contour details for the vascular endings. This demonstrates the high segmentation accuracy and strong generalization ability of our model across different datasets.

**Figure 8:**
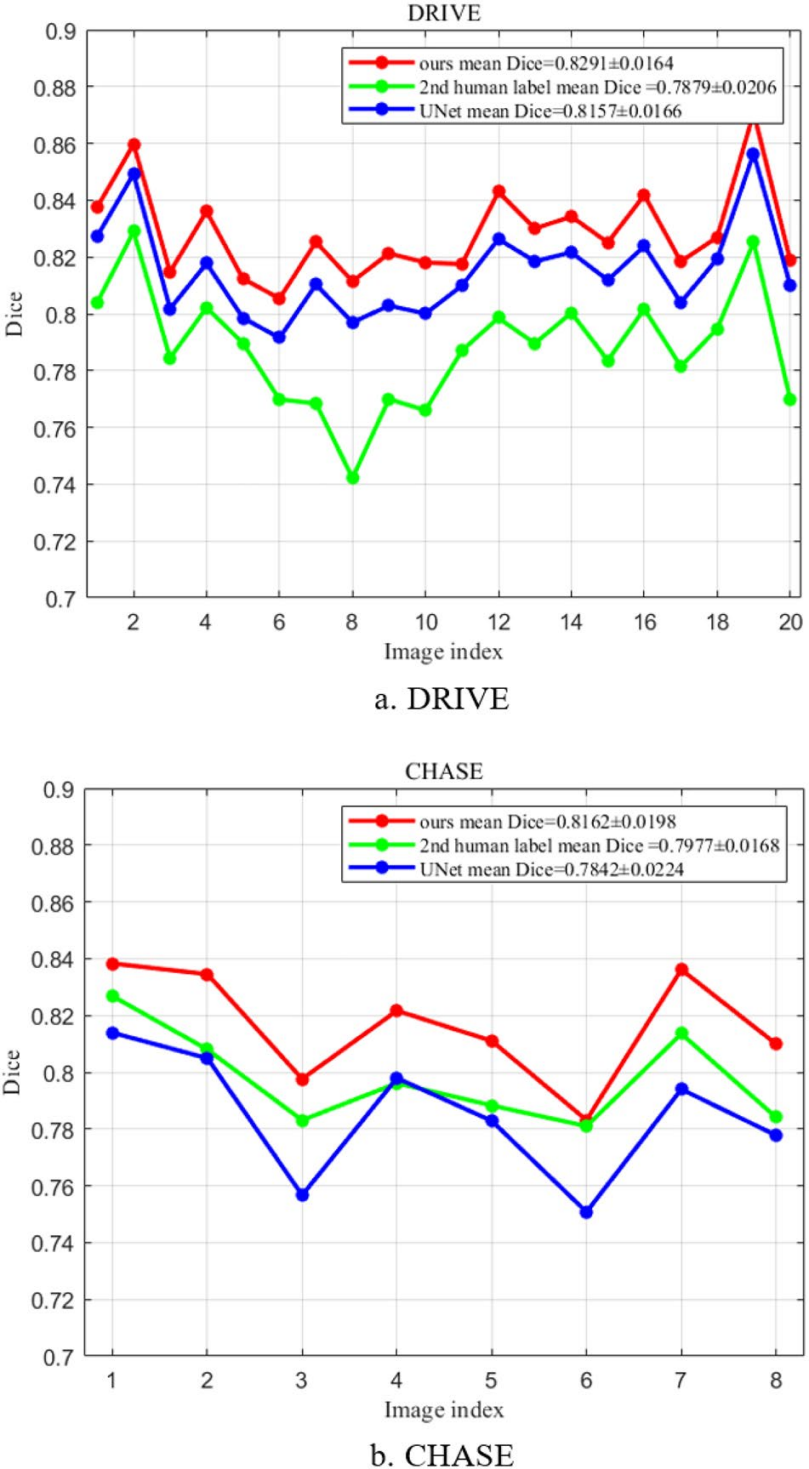
Comparison of Dice coefficient using various methods.

Figure 8 presents a comparison of the Dice coefficients for three methods: the Unet model, our model, and the manual labels provided by the second observer. Our model is trained using the manual annotations of the first expert to obtain the Dice coefficient values. The results reveal that our model outperforms both the first and second experts, achieving the highest Dice coefficient value, as depicted in Fig. 8. This comparison highlights that our model exhibits a smaller discrepancy with the labels, indicating its robustness, superior segmentation accuracy, and practical application value in comparison to the Unet model and human annotation segmentation.

**Figure 9:**
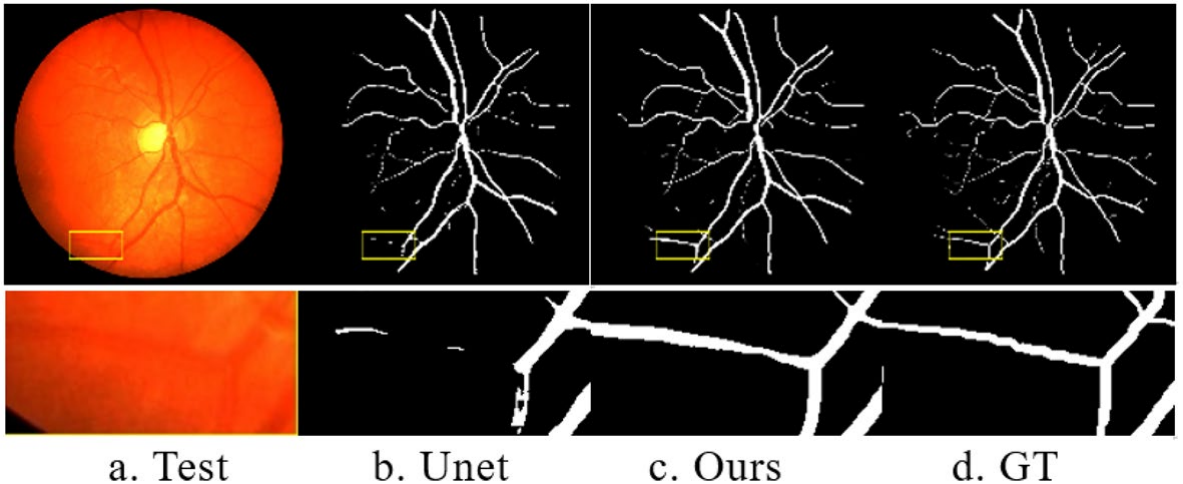
Detailed segmentation comparisons among multiple models.

Figure 9 provides a detailed comparison between our model and the Unet model, showcasing the results of the segmentation process. The enlarged local details in the second row clearly demonstrate that the Unet segmentation result exhibits more pronounced vessel ending breakages. In contrast, our model displays fewer instances of small vessel breaks, resulting in higher accuracy in segmenting vessel pixels and overall superior segmentation performance.

These advanced vessel segmentation techniques have multiple applications in patients with eye diseases. Firstly, they enable disease progression monitoring by capturing fundus images periodically and analyzing vascular changes, facilitating the determination of disease worsening and the need for treatment adjustments. Secondly, these techniques aid in the development of personalized treatment plans by providing doctors with a better understanding of the location and severity of the lesion, enabling informed decisions regarding the most appropriate treatment approach, whether it be laser treatment, drug therapy, or surgical intervention.

**Figure 10:**
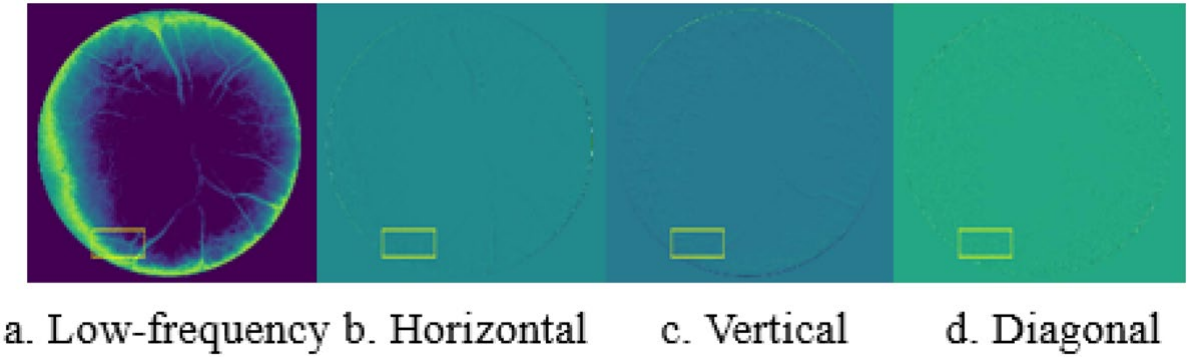
Visualization of the Haar wavelet decomposition feature map.

Figure 10 provides a visualization of the low-frequency features obtained through Haar wavelet decomposition, as well as the high-frequency features in the horizontal, vertical, and diagonal directions. It is observed that the local details in Fig. 9 are effectively represented in the high-frequency features in the vertical direction (Fig. 10c), enhancing our model’s feature extraction capability for small vessels. Furthermore, Fig. 10 also illustrates the rationality of our model.

### Ablation study

To analyze the impact of each module on our model and demonstrate the segmentation advantages of the fusion model, we conduct ablation experiments on the DRIVE^19^ dataset. The results of these experiments are presented in Table 4.

**Table 4:** Ablation experiments conducted on the DRIVE dataset.

No.	Model	Unet	SegNet	HarrNet	AE	DSL	Dice	SE	SP	Acc
1	Unet^10^	$\surd$					0.8157	0.7942	0.9826	0.9622
2	SegNet^21^		$\surd$				0.8172	0.7893	0.9792	0.9550
3	HaarNet			$\surd$			0.8178	0.8013	0.9769	0.9545
4	Unet+SegNet	$\surd$	$\surd$				0.8187	0.8010	0.9773	0.9548
5	SegNet+HaarNet		$\surd$	$\surd$			0.8242	0.7875	**0.9820**	0.9565
6	Unet+HaarNet	$\surd$		$\surd$			0.8237	0.8010	0.9790	0.9564
7	TP-Unet	$\surd$	$\surd$	$\surd$			0.8252	0.7985	0.9801	0.9569
8	TP-Unet+AE	$\surd$	$\surd$	$\surd$	$\surd$		0.8256	0.7986	0.9801	**0.9571**
9	**TP-Unet+AE+DSL**	$\surd$	$\surd$	$\surd$	$\surd$	$\surd$	**0.8291**	**0.8184**	0.9773	**0.9571**

Significant values are in bold.

Experiments No. 1–3 represent the outcomes of independent experiments using the Unet^10^, SegNet^21^, and HaarNet model, respectively. It is observed that the HaarNet model exhibits higher sensitivity and Dice coefficient, while the SegNet model demonstrates greater accuracy and specificity. On the other hand, the Unet model shows a more balanced performance across all indices.

Experiments No. 4-6 display the segmentation results of the pairwise fusion of Unet^10^, SegNet^21^, and HaarNet model. Notably, the two-two fusion model showcases an improvement of $0.64\%$ in terms of the optimal Dice coefficient compared to the independent experiments. This finding indicates that the two-two fusion model generally delivers superior segmentation performance. However, it is important to note that while HaarNet shows a higher improvement in Dice accuracy when fused with the other two models, its sensitivity remains unaffected. This suggests that direct fusion alone is insufficient to enhance the model’s segmentation ability for vascular pixels.

Experiments No. 7-9 pertain to the ablation experiments of the TP-Unet model fused with the AE block and DSL block. The TP-Unet+AE+DSL model achieves the highest Dice coefficient among all the experiments, surpassing the results of experiments No. 1-6. It is evident that the addition of the AE block contributes to improvements in each indicator. Subsequently, the fusion of the DSL block further enhances the Dice coefficient by $0.35\%$ and sensitivity by $1.98\%$. These results demonstrate the efficacy of the AE and DSL blocks in bolstering the feature fusion capability of TP-Unet and improving its segmentation performance.

Considering the accuracy of the Dice coefficient, the TP-Unet+AE+DSL model should be chosen. However, if the focus is on minimizing the number of parameters and model complexity, the TP-Unet+AE model can be utilized to achieve a smaller parameter count and reduced complexity. Nevertheless, in the context of medical imaging, where accurate segmentation is of utmost importance, prioritizing algorithmic accuracy outweighs concerns about model complexity.

### Equilibrium coefficient

In this subsection, we will examine the impact of different equilibrium coefficients of AE (auto-encoder) and DSL (deep supervised learning) blocks on the accuracy of retinal image segmentation. The experimental analysis results for this investigation are presented in Table 5, with a fixed value of $\alpha _1=1$.

**Table 5:** Analysis of equilibrium coefficients at various values.

$\alpha _i$	$\beta$	Dice	SE	SP	Acc
0.1	0.0	0.8287	0.8157	0.9777	0.9571
0.2	0.1	**0.8291**	**0.8184**	0.9773	0.9571
0.1	0.2	0.8274	0.8068	0.9767	**0.9572**
0.0	0.1	0.8256	0.7986	**0.9801**	0.9570

Significant values are in bold.

The findings clearly indicate that when $\alpha _i=0.2$ and $\beta =0.1$, the Dice coefficient and sensitivity values are higher compared to those obtained when $\alpha _i=0$ and $\beta =0.1$ or $\alpha _i=0.1$ and $\beta =0$. This suggests that the fusion of both blocks has a more favorable effect on performance than the fusion of a single block alone. Furthermore, the accuracy is improved when $\alpha _i=0.1$ and $\beta =0.2$, surpassing the results achieved with $\alpha _i=0.2$ and $\beta =0.1$. These outcomes highlight the potential for enhancing the segmentation performance of the TP-Unet+AE+DSL model by employing appropriate equilibrium coefficient values, as demonstrated in Table 5.

### Efficiency

In Table 6, in comparison with the simplified deep learning method, our model does not attain optimal efficiency in terms of parameters (Params.), memory, and floating-point operations (FLOPs.). However, when compared with Unet^10^, R2Unet^11^, our model demonstrates a reduction in the number of parameters and higher computational efficiency. This advantage stems from the utilization of only three down-sampling convolutions without 1024 convolution cores, which allows for a reduction in the parameters required for feature fusion.

**Table 6:** Performance analysis.

Model	Params.	Memory	FlOPs.
Unet^10^	40.1 M	2068 M	261.74 G
R2Unet^11^	44.7 M	2312 M	262.75 G
SegNet^21^	3.8 M	686 M	38.97 G
HaarNet	7.1 M	726 M	51.0 G
**Ours**	15.6 M	2221 M	132.58 G

Significant values are in bold.

On the other hand, our model falls behind in terms of parameters and computational efficiency when compared to SegNet^21^, HaarNet. Nevertheless, when considering the evaluation data presented in Table 4, our model achieves greater accuracy. This is attributed to the implementation of the DSL and AE modules, which are exclusively employed during the training phase and don’t impact the overall reasoning speed. However, they do contribute to enhancing the accuracy of our model.

Although the improvement may be modest, in the context of medical image segmentation, precision holds greater significance than parameters or speed. Thus, our model assumes greater significance in this domain.

## Conclusion

In order to address the issues of low accuracy and under-segmentation in existing retinal vessel segmentation methods, we propose a novel approach using a three-path feature fusion model. This model combines the strengths of the Unet, SegNet, and HaarNet model. Additionally, we incorporate the advantages of the AE block and DSL block to construct the TP-Unet+AE+DSL model framework.

Through experimental simulations, we have observed that our proposed model achieves high evaluation index values and produces visually appealing segmentation results. Furthermore, our ablation experiments demonstrate that the multi-path fusion approach yields higher segmentation accuracy compared to using a single path. The effectiveness of the AE and DSL blocks in optimizing the TP-Unet model has also been verified. Moreover, we have discussed the impact of different balance coefficients on the evaluation metrics of our model.

However, it is important to note that the integration of multiple paths, the AE and DSL blocks increase the number of parameters and computational complexity. In future research, we intend to focus on developing lightweight medical image segmentation models to mitigate these challenges.

In conclusion, our study presents a promising approach to improve retinal vessel segmentation, offering potential for more accurate and reliable results in medical image analysis.

## Data Availability

All of the datasets utilized in this paper are publicly available datasets, namely the DRIVE^19^ dataset and the CHASE^20^ dataset.
